# Efficacy of ‘Itrifal Saghir’, a combination of three medicinal plants in the treatment of obesity; A randomized controlled trial

**DOI:** 10.1186/2008-2231-20-33

**Published:** 2012-09-10

**Authors:** Seyed Hamid Kamali, Ali Reza Khalaj, Shirin Hasani-Ranjbar, Mohammad Mehdi Esfehani, Mohammad Kamalinejad, Omidmalayeri Soheil, Seyed Ali Kamali

**Affiliations:** 1 Student of Department Iranian Traditional Medicine, Faculty of Medicine, Shahed University, Tehran, Iran; 2 School of Traditional Medicines, Tehran University of Medical Sciences, Tehran, Iran; 3 Department of surgery, Mostafa Khomeyni Hospital, Shahed University, No 17. Clinic Salamat, Iranian Traditional Medicine Group, Dashtestan 3rd St., Pasdaran Ave, 1947948613, Tehran, Iran; 4 Endocrinology and Metabolism Research Institute, and Faculty of Medicine, Tehran University of Medical Sciences, 5th floor, Shariati Hospital, North Kargar Ave., Tehran, Iran; 5 Department of Pharmacognosy, School of Pharmacy, Shahid Beheshti University of Medical Sciences, Tehran, Iran; 6 Khatam Hospital, Tehran, Iran

**Keywords:** Obesity, Itrifal Saghir, Traditional Iranian medicine, Randomized trial, Terminalia chebula, Terminalia bellerica, Emblica officinalis, Triphala

## Abstract

**Background:**

Herbal combination of *Itrifal Saghir* (triphala) has been widely used in traditional medicine. And brings health benefits such as antioxidant effect and scavenger of hydroxyl radicals and nitric oxide radicals activity and substantiated in traditional medicine a anti-obesity.

**Material and method:**

In this study we aimed to assess the efficacy of this herbal medicinal on reduction of weight and body mass index (BMI) of simple obese subjects in comparison with placebo. Obese subjects aged between 16 and 60 years were selected for 12-week, double-blind, randomized, placebo-controlled trial using a parallel design. Subjects were randomly assigned to take 5 grams of either the Itrifal Saghir (*n* = 31) or placebo (*n* = 31), 2 times daily for 12 weeks. Measures of body weight, BMI, waist circumference (WC), hip circumference (HC), were assessed at baseline and once every four weeks during the 12 week treatment period. The safety was evaluated by means of measuring the liver and kidney function. Homeostasis model of insulin resistance (HOMA-IR) was calculated as [fasting insulin (μU/mL) × fasting glucose (mmol/L)/22.5].

**Results:**

Compared to placebo group, in treatment group the mean difference of effective weight loss was 4.82Kg (CI95% 3.52 - 6.11, ρ < 0.001), the mean of decrease in waist circumference was 4.01 cm (CI 95% 2.13 - 5.90, ρ < 0.001), and the mean decrease in hip circumference was 3. 21 cm (CI 95% 1.96 - 4.45, ρ < 0.001) in treated subjects. No adverse effects or significant changes in liver and kidney function tests were observed in both placebo and treated groups.

**Conclusions:**

Itrifal Saghir appears to produce a positive effect on weight loss in obese subjects.

## Background

Obesity is recognized as an important metabolic disorder and a major public health issue which affects a large number of people around the world. According to the World Health Organization (WHO), obesity is rated among the 10 most preventable disease of clinical and public health [1]. Obesity is associated with an array of health problems in adult and pediatric population including metabolic syndrome [2-5], hypertension, diabetes [2], and liver disease [5-8]. In human, adipose tissue represents an active endocrine organ that by releasing the large number of bioactive mediators plays an important role in modulating hemostasis, blood pressure, lipid and glucose metabolism, and inflammation. It has been shown that weight loss as modest as 10% could significantly improve the risk of several chronic diseases [3,9,10]. Obese people have very high levels of oxidative stress, caused by a build-up of free radicals [5], and obesity is known to be one of the conditions that decrease antioxidant capacity [11,12].

It is assumed that obesity decreases antioxidant defense by lowering the circulatory levels of antioxidant enzymes such as catalase, glutathione peroxidase and glutathione reductase [12]. A number of studies have demonstrated that antioxidants may act as a regulator of obesity in animal [13,14]. Complementary therapies recently are being used increasingly worldwide. When conventional medicine fails to treat chronic diseases efficaciously and without adverse events, many people seek unconventional therapies including herbal medicine [15]. Many herbal medicinal products have been used for several decades or even hundreds of years. One of this traditional products, Itrifal Saghir (etrifel saqir) or atrifal arabized of Triphala [16] is a polyherbal preparation that has been marketed in Iran for a several years. It composed of the three medicinal fruits Phyllanthus emblica L. *or* Emblica officinalis Gaertn*. (Euphorbiaceae),* Terminalia chebula Retz*. (Combretaceae), and* Terminalia belerica Retz*. (Combretaceae)* and formulated for the treatment and management of obesity. It is also claimed that Itrifal Saghir produces beneficial effects on heath and helps the digestive tract to work at optimal level [17,18]. In Al-Qanun fit-tib, the Itrifal Saghir was prescribed for the relaxation of the stomach, its moisture [17,18], a obviation of obesity [17,19,20]. The ingredients presented in Itrifal Saghir are known to have natural antioxidant activity and scavenger of hydroxyl radicals and nitric oxide radicals activity, anti-inflammatory, anti-hypercholesterolemic [20]. To date, there have been only two published animal trials evaluating the weight loss effects of Itrifal Saghir [21,22]. However, there is lack of detailed trials on human subjects. In this regard, we aimed to conduct a randomized, placebo-controlled clinical trial to evaluate the weight reducing effects and safety of 3 months consumption of commonly used dose of Itrifal Saghir (10 g/day) in healthy adult subjects being on a typical Iranian diet.

## Method and material

### Study design

The study was designed as a prospective, two-arm, randomized, double-blind, placebo-control study and using a parallel design. Sixty-two patient were randomly assigned into two groups of placebo (n = 31) and treated (n = 31). Patients received either 10 g of Itrifal Saghir/day or placebo for 3-months and were followed for clinical efficacy at weeks 1, 4, 8 and 12. Randomization of equal numbers of subjects to placebo or treated group was achieved using a random number table, with block sizes varying between two and eight. A statistician, who was not involved in the study, produced separate randomization codes for the two sides ensuring the balance of gender, age, and severity of obesity between groups. Sealed copies of these codes were provided to the investigators for emergency identification. Subjects who left the study prior to the first post-randomization visit were replaced with subjects assigned to the same treatment condition in the study. Codes were remained sealed until completion of the study. The analysis was performed by who was blinded to the nature of the study.

### Study population

The participants were recruited from the outdoor patients who referred to obesity clinic of Mostafa Khomeini hospital, Tehran, Iran. The subjects were informed to participate in the study by flyers placed in an area close to the hospital. Subjects were selected according to our defined inclusion criteria which was: age 16–60 years, a body mass index (BMI) between 30 to 50 kg / m^2^. For subjects aged between 50 to 60 years, having a normal electrocardiography was mandatory. Our exclusion criteria were defined as: heavy smokers (more than twenty cigarettes per day); alcohol consumption; pregnancy; recent surgery, patients with coronary heart disease; known cases of diabetes mellitus; patients with proven malignancy, asthma, chronic cough, chronic inflammatory disease and psychological problems; history of chronic kidney and liver disease, obesity due to endocrine disease (hypothyroidism or uncontrolled thyroid disease); and genetic obesity syndrome. Indeed patients who were treated with sliming drugs or taking the diet regimen in the last 6 months and those receiving corticosteroid or immunosuppressive medications were excluded from study. The study had the approval of the local ethics committee of Shahed University with reference number of 112251/4 and was conducted in accordance with the Helsinki Declaration (1983 version, and trial registration ID in IRCT: IRCT201104206237N1). Subjects were given written information and a verbal explanation concerning the study prior to obtaining consent for their participation. All the participants signed a written consent form before recruiting in the study.

The subjects were not allowed to receive other obesity management and were asked to keep to their existing diet and life style during the study period. All subjects were free to withdraw at any time during the course of the study.

### Intervention

Fruits of Terminalia chebula Retz. (Family combretaceae) [Popular Marked Plant (PMP)-622, PMP-623, PMP-624], Terminalia bellerica Roxb (Family combretaceae) (PMP-625). (Combretaceae) and Phyllanthus emblica L. (Family euphorbiaceae) (PMP-626), were purchased from local market and authenticated at Botanical of Traditional Medicines School of Tehran University of Medical Sciences. A voucher specimen was preserved in our laboratory for future reference. Seeds from individual fruits were removed and the dried fruit pulp was crushed to powder using a grinder. Itrifal Saghir was prepared from these powders by mixing them in equal proportions (1:1:1) based on Al-Qanun fit-tib Formula [19] and main traditional medicine books (such as eksir azam and tebbe akbari) [18]. The powder was weighted and these ingredients should be mixed with almond oil and then kneaded with froth less honey. These were stored in a closed vessel for future use and packed in boxes. Subjects in treated group were required to take 5 gram (one tea spoon) of majoune (confection), two times per day (one before breakfast and one after dinner) for three months. The placebo group received an identical appearance herbal confection [ACRPOL 934P (Carbomer 943P)] that contained no active substances and has been approved to be safe for human consumption (Additional file 1: Appendix 1) [23,24]. The placebo was purchased from akbariyeh corporation (agent of corel pharma chem). To ensure the compliance of patients, the subjects were asked to return the given box in each follow-up visit. The box was weighted and the amount of used confection was regarded as a measure of drug consumption. Patient who missed more than 20% of the total dose of prescribed treatment was considered non-compliance and excluded from study.

### Measurements

All the participants underwent physical examination and anthropometric evaluations at the time of recruitment and follow-up visits. By means of a special questionnaire, the demographic information and medical history were collected. At each visit participants were also queried about changes in medications and adverse events. Height was measured with a wall-mounted Stadiometer and weight was measured while wearing light clothes without shoes on a calibrated balance beam scale with error of nearest ± 0.1 kg (Gmbh & co.kg. Germany. Ser. no. 5769/59092207. Model 769/32/994). BMI was calculated according to the formula: BMI = body weight (BW) / squared height (kg / m^2^).

Waist circumference was measured at the end of a normal expiration at the midpoint between the lower margin of the last palpable rib and the top of the iliac crest (hip bone). Hip circumference was measured at the maximum circumference over the buttocks; by soft, non-stretchable plastic tape. Blood pressure was measured two times (with a 5 minutes interval) using a standard calibrated mercury sphygmomanometer on the right hand after the participants had been sitting for at least 15 minutes. The mean of two measurements were recorded as the blood pressure. Heart rate and temperature also were recorded. Diet stability was verified using the 24-hour diet recalls at baseline and end of the study. Blood collections for serological data were conducted only at the first and the last visit (week 12). The peripheral blood samples were obtained following 8 hours overnight fasting. All measurements were performed at the laboratory of endocrine and metabolism research center of Tehran University of Medical Sciences.

The serological data included cholesterol, low density lipoprotein cholesterol (LDL), high-density lipoprotein cholesterol (HDL), triglycerides, fasting insulin and glucose (FBS), glycosylated hemoglobin (HbA1c), Prothrombin time (PT), and renal functions tests such as creatinine, and liver functions tests such as aspartate transaminase (AST), alanine transaminase (ALT) and alkaline phosphatase (ALP) tests and other tests such as uric acid. All the measurements were carried out according to standardized established method (Additional file 1: Appendix 2).

The related adverse effects were assessed based on the self- reports symptoms and also answering a questioning and complete physical examination at each visit. Filling the questionnaire was performed by a trained physician who was blind to participant’s group.

### Statistical analyses

Statistical Package for the Social Sciences (SPSS) software was used for all statistical analysis [25]. Results are reported as the mean ± standard deviation. Normality of continuous variable was assessed by Kolmogorov-Smirnov. Chi-square test was used for comparison of the frequency of variables between two groups. Student T-test and general Linear Model, Such as univariate tests, multivariate tests, mauchly's test of sphericity, pairwise comparisons were used to compare quantitative variables. P values less than 0.05 were considered to be statistically significant.

## Results

**Figure 1:**
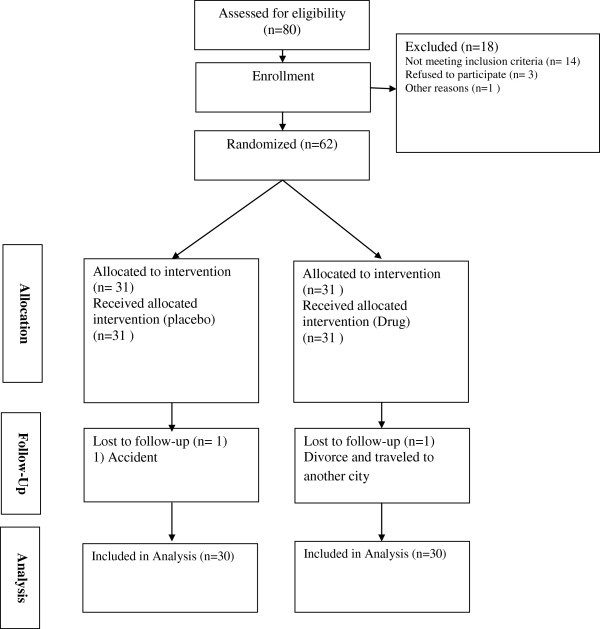
The trial flowchart.

A total of 62 subjects were randomly assigned into two groups, treated and placebo group (31 patients in each group). Age of the patients ranged from 16 to 60 years. No significant difference in regards to age, gender, level of education, marital status, and waist circumference was observed between two groups (Table 1). Indeed the rate of drop out was similar in both groups (Figure 1). Two groups showed similar treatment compliance (variation between 92% and 99%) after 3 months of follow-up.

**Table 1:** Base Line characteristics of the subjects

**Title**	**Intervention group**	**Placebo group**	**ρ-value**
	Number (%)	Number (%)	
literacy	Under diploma = 15 (50%)	Under diploma = 14 (46.66%)	0.80
	Upper diploama = 15 (50%)	Upper diploama = 16 (53.33%)	
Sex	Male = 6 (20%)	Male = 8 (26.66%)	0.54
	Female = 24 (80%)	Female = 22 (73.33%)	
Marital status	Single = 9 (30%)	Single = 10 (33.33%)	0.78
	Marriage = 21(70%)	Marriage = 20 (66.66%)	
Age(year)*	39.16 ± 9. 59	36.36 ± 9. 9	0.27
Height(cm) *	161.18 ± 6.63	163.15 ± 7.20	0.27
Weight(kg)*	96.89 ± 14.07	96.56 ± 13.39	.92
BMI( kg/m^2^)*	37.14 ± 5.40	36.29 ± 4.66	.52
WC (cm)*	115.96 ± 13.68	113.86 ± 10.60	.50
HC (cm)*	122.40 ± 10.56	119.40 ± 8.87	.23

* mean, and standard deviation.

Abbreviations: *BMI*: body mass index; *WC*: Waist circumference; *HC*: Hip circumference.

Itrifal Saghir was generally well tolerated and no remarkable adverse events were reported in treated group. The rate of reported minor side effects was similar in both groups.

Comparing mean differences between group significant decline in weight, waist and hip circumferences, and BMI (ρ <0.001) were found after 12 weeks of treatment, while no remarkable changes in these variables were noticed in placebo group as shown in Table 2. Moreover, mean difference of effective weight loss was 4.82Kg (CI95% 3.52 - 6.11, ρ < 0.001), such as the mean difference of effective waist circumference decrease was 4.01 cm (CI 95% 2.13 - 5.90, ρ < 0.001), and the mean decrease in hip circumference was 3.20 cm (CI 95% 1.96 - 4.45, ρ < 0.001). Compared to placebo group no remarkable changes in these variables were noticed.

**Table 2:** The effect of the Itrifal Saghir administration on antropometric parameters and blood pressure

**Title**	**Intervention group**		**Placebo group**		P-value**
	**Mean ± SD**		**Mean ± SD**		
	**Start treatment**	**End treatment**	**Start treatment**	**End treatment**	
weight (Kg)	96.89 ±14.07	92.52 ± 14.44	96.56 ± 13.39	97.01 ± 13.49	>0.001
BMI ( g/m^2^)	37.14 ± 5.40	35.67 ± 5.48	36.29 ± 4.66	36.47 ± 4.74	>0.001
WC (cm)	115.96 ± 13.68	112.43 ± 13.99	113.86 ± 10.60	114.36 ± 11.19	>0.001
HC (cm)	122.40 ±10.56	119.50 ± 10.32	119.40 ± 8.87	119.83 ± 8.91	>0.001

**mean differences P-value.

Abbreviations: *BMI*: body mass index; *WC*: Waist circumference; *HC*: Hip circumference; *NS*: Not statistically significant.

The mean of fasting blood sugar increased from 94.72 ± 8.96 to 99.20 ±10.55 in the placebo group and decreased from 96.86 ± 12.16 to 94.20 ± 9.78 in the Itrifal Saghir group after 12 weeks (ρ = 0.006). The mean fasting serum insulin decreased from 18.46 ± 10.91 to14.76 ± 8.00 in the Itrifal Saghir group and increased from 18.24 ± 7.21 to 19.91 ± 8.55 in the placebo group after 12 weeks (ρ = 0.01).

The mean value of ALT decreased from 24.10 ± 9.18 to 18.90 ± 8.07 in the Itrifal Saghir group that was statistically significant comparing to placebo group (ρ = .003). And the mean of uric acid decreased from 6.09 ± 1.07 to 5.78 ± 1.17 in the Itrifal Saghir group that was statistically significant comparing to placebo group (ρ = .05). Other Serological characteristics are shown in Table 3.

**Table 3:** The effect of the Itrifal Saghir administration on laboratory tests

**Title**	**Intervention group**		**Placebo group**		P-value**
	**Start treatment**	**End treatment**	**Start treatment**	**End treatment**	
	**Mean ± SD**	**Mean ± SD**	**Mean ± SD**	**Mean ± SD**	
FBS (mg/dL)	96.86 ± 12.16	94.20 ± 9.78	94.72 ± 8.96	99.20 ± 10.55	.006
GTT(120 min) (mg/dL)	113.66 ± 31.58	111.20 ± 32.082	113.28 ± 33.79	121.92 ± 35.70	.09
HbA1c %	4.40 ± .71	5.26 ± .70	4.57 ± .94	4.63 ± .95	>0.001
CREATININE(mg/dL)	.92 ± .12	1.0 ± . 15	.91 ± .16	.94 ± .17	.15
URIC ACID(mg/dL)	6.09 ± 1.07	5.78 ± 1.17	6.36 ± 1.53	6.47 ± 1.32	.05
TRIGLYCERIDES(mg/dL)	118.76 ± 57.72	114.00 ± 47.68	181.41 ± 159.57	181.17 ± 160.93	.41
CHOLESTROL (mg/dL)	186.80 ± 38.40	178.86 ± 32.66	206.31 ± 30.93	201.20 ± 37.32	.26
HDL(mg/dL)	59.50 ± 10.47	53.33 ± 9.27	52.79 ± 8.78	50.44 ± 10.85	.54
LDL(mg/dL)	101.46 ± 27.19	102.50 ± 25.66	110.39 ± 34.015	103.60 ± 36.53	.04
AST (u/l)	19.73 ± 4.90	18.50 ± 5.88	20.86 ± 8.19	21.10 ± 8.24	.18
ALT (u/l)	24.10 ± 9.18	18.90 ± 8.07	29.06 ± 15.69	29.44 ± 16.98	.003
ALP (u/l)	197.93 ± 50.49	199.40 ± 48.86	182.75 ± 37.13	184.27 ± 41.50	.77
WBC (1000/μl)	6.52 ± 1.67	6.32 ± 1.89	6.38 ± 1.07	6.47 ± 1.21	.42
RBC(mil/ μl)	4.82 ± .32	4.82 ± .39	4.88 ± .50	4.87 ± .50	.99
HEMOGLOBIN (g/dl)	13.45 ± 1.23	13.87 ± 1.54	13.76 ± 1.51	13.95 ± 1.49	.28
HEMATOCRIT(%)	41.29 ± 2.99	42.16 ± 3.89	41.66 ± 3.39	42.287 ± 3.93	.75
MCV(fl)	85.60 ± 4.22	87.48 ± 5.06	85.62 ± 4.52	86.95 ± 4.00	.39
MCH(pg)	28.91 ± 6.26	28.78 ± 2.50	28.25 ± 1.87	28.71 ± 1.95	.85
MCHC(%)	32.56 ± 1.39	32.86 ± 1.43	33.05 ± 2.51	33.01 ± 2.25	.60
PLATELETS(1000/μl)	265.60 ± 57.50	264.26 ± 66.44	250.03 ± 44.60	254.68 ± 42.48	.57
PT	12.87 ± . 52	12.74 ± . 53	12.80 ± . 49	12.9 ± .46	.14
INR	1.05 ± .06	1.03 ± .06	1.05 ± .07	1.04 ± .06	.40
Fasting serum insulin( mIU/mL)	18.46 ± 10.91	14.76 ± 8.00	18.24 ± 7.21	19.91 ± 8.55	.01

**mean differences P-value.

Abbreviations: *LDL*: low density lipoprotein cholesterol; *HDL*: high-density lipoprotein cholesterol; *FBS*: fasting insulin and glucose; *GTT*: glucose tolerance test; *HbA1c*: glycosylated hemoglobin; *PT*: Prothrombin time; *AST*: aspartate transaminase; *ALT*: alanine transaminase; *ALP*: alkaline phosphatase; *WBC*: white blood cells; *RBC*: red blood cells; *INR*: International Normalized Ratio; *MCV*: Mean corpuscular volume; *MCH*: mean corpuscular hemoglobin; *MCHC*: mean corpuscular hemoglobin concentration; *NS*: Not statistically significant.

The comparing between start and end of systolic blood pressure level from 119.66 ± 17.80 mmHg and 117.50 ± 18.03 mmHg (with five minute interval) at baseline to 120.22 ± 20.08 mmHg and 119.77 ± 18.61 mmHg at 12 week and the mean diastolic blood pressure level from 78.83 ± 10.05 mmHg and 78.83 ± 10.56 mmHg at baseline to 80.00 ± 12.14 mmHg and 79.77 ± 10.74 mmHg at week 12 in intervention group and the mean systolic blood pressure level from 122.00 ± 16.22 mmHg and 121.16 ± 14.66 mmHg (with five minute Interval) at baseline to 121.15 ± 20.52 mmHg and 122.30 ± 23.05 mmHg at 12 week and the mean diastolic blood pressure level from 81.66 ± 9.31 mmHg and 81.66 ± 9.49 mmHg at baseline to 78.07 ± 10.31 mmHg and 80.38 ± 11.98 mmHg at 12 week in placebo group, independent samples t-test revealed changes were not statistically significant in both groups.

### Safety and tolerability

There were no significant changes in renal function or in levels of liver-associated enzymes among any of the treatment groups. Itrifal Saghir was generally well tolerated without statistically significant differences in rates of any adverse events.

## Discussion

The present study evaluated weight reduction, a combination of three herbal remedies that are traditionally known for their weight reduction effects. In traditional medicine, mild overweight is generally accepted while severe obesity is considered a suitable condition to be treated using specific medicinal plants, body exercises and food consumption control. The Itrifal Saghir combination contained Seedless plants of: Terminalia chebula Retz., Terminalia belerica Roxb., and Emblica officinalis l. in equal parts. These ingredients should be mixed with almond oil and then kneaded with froth less honey and be kept in the vessel for further use [17]. In this study, Itrifal Saghir was given as a supplement at the levels of 10 g/day for a period of 12 weeks. The range of doses used in the present study was consistent with those in Al-Qanun fit-tib [18] in which the useful effects of Itrifal Saghir in obviation of obesity were suggested [17,19]. To our knowledge no study has been performed yet to evaluate the effects of this herbal compound on body weight and BMI, however in animal studies a reduction of body has been reported by El-Mekkawey et al., and Somasundram et al., [21,22]. suggested mechanism in some herbal effective in treatment currently available anti-obesity medications attack the body fat dilemma in three different ways. They can stimulate metabolism, suppress appetite, affect serotonin, or they can impede digestion of fat [26]. Oxidative stress plays critical roles in the pathogenesis of various diseases [27]. It was shown that obesity may induce systemic oxidative stress and increased oxidative stress in accumulated fat is one of the underlying causes of dysregulation of adipocytokines [5]. Moreover obesity has been shown to be one of the conditions that decrease the antioxidant capacity [14,28]. The Itrifal Saghir has a good abundance of antioxidant, which may explain the useful effects of this compound in the treatment of high oxidative stress condition such as obesity [28]. In fact the antioxidant potential of various Itrifal Saghir formulations have been evaluated in various studies [20]. Itrifal Saghir is reported to be effective in free radical scavenging and decreasing oxidative stress [28] and inflammation [29]. In another study of a randomized, double blind placebo controlled trial showed that the LDL/HDL cholesterol ratio significantly declined; fasting and postprandial blood glucose as well as lipid peroxides and diene conjugates (indicators of oxidative stress) significantly decreased in the triphala group treatment [30]. High-performance liquid chromatography analysis showed that Itrifal Saghir contains gallic acid as the major component [31]. Gallic acid (3,4,5-trihydroxybenzoic acid; GA) is a naturally abundant phenolic compound [32]. It is reported to have antioxidant activity and is expected to reduce the risk of disease and brings health benefits through daily intake [33]. Taking together all these observations point to an antioxidan effect and stimulate metabolism, suppress appetite, affect serotonin, or they can impede digestion of fat and decreased fasting and postprandial blood glucose and LDL/HDL cholesterol and free radical scavenging and decreasing oxidative stress of Itrifal Saghir and one may assume that the beneficial effect of Itrifal Saghir on weight reduction seen in this study might be mediated through this property. However further study for evaluation of mechanism involved in this process is highly suggested. Itrifal Saghir in its compound form acts more efficiently most probably than the individual components [18,34]. The safety of Itrifal Saghir consumption was evaluated in previous pre-clinical studies using LD50, MTT and LDH assays [35]. These researches showed no toxic effects of Itrifal Saghir in doses up to 240 mg/kg. The intra peritoneal LD50 dose of Itrifal Saghir was found to be 280 mg/kg [36]. Another study showed the aqueous and alcoholic extracts of Itrifal Saghir up to a dose of 1750 mg/kg were considered safe when evaluated for acute oral toxicity performed in accordance with the Organization for Economic Cooperation and Development guidelines [37]. Indeed no drug-induced mortality has been reported [36].

We need to mention some limitations that we have faced in this study. These limitations are mostly common in studies involving human subjects. First, the traditional medicine have two main variable: one which exist in human including mezaje (temperament) and racial/ethnic, sex, age, territory, season, occupation and second is the herbal drugs which are natural products and their chemical composition, therefore, varies depending on several factors, such as geographical source of the plant material, climate in which it was grown, time of harvest, etc. These variations can result in bioavailability differences in humans. Indeed in this study our subjects were assessed only for 12 weeks, therefore we could not have any conclusion about long term efficacy of this compound. Another, we did not recommend a specific diet in this study.

## Conclusion

The Itrifal Saghir brought a significantly greater weight loss than placebo during the study period in obese individuals. This was accompanied by a significant improvement in the waist and hip circumference. Itrifal Saghir possesses antioxidant and free radical scavenging and anti hyperlipidemia and lower fasting properties that could have applications in metabolic as well as other physiological complications in which there is an increase in oxidative stress. These new findings warrant further exploration into the active compound of Itrifal Saghir and the potential of its newly discovered weight loss benefits. However further study for evaluation of mechanism involved in this process is highly suggested.

## Abbreviations

BMI: Body mass index; WC: Waist circumference; HC: Hip circumference; LDL: Low density lipoprotein cholesterol; HDL: High-density lipoprotein cholesterol; FBS: Fasting blood glucose; GTT: Glucose tolerance test; HbA1c: Glycosylated hemoglobin; PT: Prothrombin time; AST: Aspartate transaminase; ALT: Alanine transaminase; ALP: Alkaline phosphatase; Homa: Homeostasis model assessment; WBC: White blood cells; RBC: Red blood cells; INR: International Normalized Ratio; MCV: Mean corpuscular volume; MCH: Mean corpuscular hemoglobin; MCHC: Mean corpuscular hemoglobin concentration; NS: Not statistically significant.

## Competing interests

The authors declare that they have no competing interests.

## Authors’ contributions

SHK participated involved have made substantial contributions to conception and design, in the sequence alignment, acquisition of data, and analysis and interpretation of data, and drafting the manuscript and have given final approval of the version to be publish. KAR participated involved in revising. SHR participated involved in design, interpretation of data, and revising, have given final approval of the version to be publish. MME participated involved in revising. MK participated involved in revising. SAK participated involved have made substantial contributions to conception. OS participated involved have made substantial contributions to conception. All authors read and approved the final manuscript.

## Supplementary Material

Additional file 1 **Appendix 1. **Carbomer 934P. **Appendix 2. **Table of standard laboratory test. Click here for file
