# Supplementary material for: Efficacy of ‘Itrifal Saghir’, a combination of three medicinal plants in the treatment of obesity; A randomized controlled trial
Source: Daru. 2012 Sep 10;20(1):33. doi: 10.1186/2008-2231-20-33 (PMC3559014; doi:10.1186/2008-2231-20-33)
Supplement: Additional file 1 — Appendix 1. Carbomer 934P. Appendix 2. Table of standard laboratory test. [file 2008-2231-20-33-S1.docx]

Appendix (1)

Carbomer 934P

| Grade & Applications | | | | |
| --- | --- | --- | --- | --- |
| Grades | | Solvent used in process | | |
| Carbomer 934P (Benzene free) | | Benzene Cyclohexane & ethyl acetate | | |
| Carbomer 934P - High purity oral pharmaceutical grade for formulations. | | | | |
| Certificate of Analysis ( CARBOMER - 934P ) | | | | |
| Batch no: 22101015 ; DATE: 08/01/2011; A. R. NO.: CRL / FP / 015 / 10-11; MFG. MONTH: DEC.- 2010 | | | | |
| No. | Test | | Specification | Results |
| 1 | Description | | White Free Flowing Powder | White Free Flowing Powder |
| 2 | Solubility | | When neutralize with alkali hydroxide or amine it dissolve in water alcohol and glycerin | When neutralize with alkali hydroxide or amine it dissolve in water alcohol and glycerin |
| 3 | Identification | | 1. (1).1% dispersion with thymol blue orange colour  (2). 1% dispersion with thymol red yellow colour 2. Very viscous gel produce | orange colour  yellow colour  Very viscous gel produce |
| 4 | Viscosity (0.5% Solution) | | Bet'n 29400 To 39400 Cps | 31,000 Cps |
| 5 | Loss on Drying | | NMT 2.0% | 0.79% |
| 6 | Heavy Metals | | NMT 0.002% | <0.0002% |
| 7 | Limit Of Benzene (By Gc) | | NMT 0.01% | Nil |
| 8 | Organic Volatile Impurities | | As per USP | Complies |
| 9 | Assay For Carboxylic Acid Content | | Bet'n 56.0% To 68.0% | 59.9% |

**Appendix (2)**

**Table of standard laboratory test**

| Premium biolis 24i- Tokyo BOEKI | | Autoanalyser |
| --- | --- | --- |
| Pars azmoon(Diasis) | Control: Trulab N, P | Calibrator: Trucal |
| Kit | Method | Test |
| Pars azmoon | GOD_PAP | Glucose |
| Man | Colorimetric Jaffe, Kinetic | Creatinin |
| Pars azmoon | CHOD_PAP | Cholesterol |
| Pars azmoon | GPO-PAP | Triglycerid |
| Pars azmoon | Direct-photometry | High-density lipoprotein cholesterol |
| Pars azmoon | Direct-photometry | Low density lipoprotein cholesterol |
| Pars azmoon | TOOS | Uric acid |
| Pars azmoon | IFCC Without P5P 37 C | Aspartate transaminase |
| Pars azmoon | IFCC Without P5P 37 C | Alanine transaminase |
| Pars azmoon | DGKC 1970 37C | Alkaline phosphatase |
| Pars azmoon | Immunoturbidometry | Glycosylated hemoglobin; |
| Monobind | ELISA | Insuline |
| Sysmex KX21-N | Cell counter | Complete blood cells |
| Fisher Diagnosis | Manual | Prothrombin time |

| ρ-value | Placebo group | Intervention group | Title |
| --- | --- | --- | --- |
|  | Number (%) | Number (%) |  |
| 0.80 | Under diploma = 14 (46.66%) | Under diploma = 15 (50%) | literacy |
|  | Upper diploama = 16 (53.33%) | Upper diploama = 15 (50%) |  |
| 0.54 | Male = 8 (26.66%) | Male = 6 (20%) | Sex |
|  | Female = 22 (73.33%) | Female = 24 (80%) |  |
| 0.78 | Single = 10 (33.33%) | Single = 9 (30%) | Marital status |
|  | Marriage = 20 (66.66%) | Marriage = 21(70%) |  |
| 0.27 | 36.36 ± 9. 9 | 39.16 ± 9. 59 | Age(year)* |
| 0.27 | 163.15 ± 7.20 | 161.18 ± 6.63 | Height(cm) * |
| .92 | 96.56 ± 13.39 | 96.89 ± 14.07 | Weight(kg)* |
| .52 | 36.29 ± 4.66 | 37.14 ± 5.40 | BMI( kg/m^2^)* |
| .50 | 113.86 ± 10.60 | 115.96 ± 13.68 | WC (cm)* |
| .23 | 119.40 ± 8.87 | 122.40 ± 10.56 | HC (cm)* |
| * mean, and standard deviation | | | |
| Abbreviations: BMI: body mass index; WC: Waist circumference; HC: Hip circumference | | | |
